# A Positive Role of Negative Mood on Creativity: The Opportunity in the Crisis of the COVID-19 Epidemic

**DOI:** 10.3389/fpsyg.2020.600837

**Published:** 2021-01-20

**Authors:** Ying Du, Yilong Yang, Xuewei Wang, Cong Xie, Chunyu Liu, Weiping Hu, Yadan Li

**Affiliations:** ^1^Ministry of Education Key Laboratory of Modern Teaching Technology, Shaanxi Normal University, Xi’an, China; ^2^Research Center for Linguistics and Applied Linguistics, Xi’an International Studies University, Xi’an, China; ^3^School of English Studies, Xi’an International Studies University, Xi’an, China; ^4^Shaanxi Normal University Branch, Collaborative Innovation Center of Assessment Toward Basic Education Quality at Beijing Normal University, Xi’an, China

**Keywords:** negative mood, self-focused attention, cognitive creativity, emotional creativity, COVID-19

## Abstract

The COVID-19 epidemic is associated with negative mood, which has the potential to be a powerful driver of creativity. However, the influence of negative mood on cognitive creativity and emotional creativity remains elusive. Previous research has indicated that self-focused attention is likely to be related to both negative mood and creativity. The current study introduced two self-focused attention variables (i.e., rumination, reflection) to explore how negative mood might contribute to cognitive creativity and emotional creativity. Based on a sample of 351 participants, our study found that (1) negative mood during the outbreak of COVID-19 was associated with cognitive creativity and emotional creativity. Meanwhile, there were significant serial mediation effects of rumination and reflection in the relationship between negative mood and creativity and (2) the psychological impact after exposure to the COVID-19 epidemic was positively correlated with emotional creativity but not with cognitive creativity. These results suggested that individuals, in real life and work, could achieve better creative performance through moderate self-focus. Moreover, individuals with different mood states can be induced to enhance their creativity in times of crisis through intervention training to promote reflection.

## Introduction

Creativity has long been of great interest in a wide range of fields, especially in the arts, science, and engineering (Liu et al., 2018). People have the ability to exert cognitive control over creativity (Beaty et al., 2014; Kenett et al., 2018). However, our ability to control creativity is not consistent at all times, especially in the face of sudden changes of circumstances. The COVID-19 infection caused by a novel coronavirus that emerged at the end of 2019 has now become a pandemic announced by the World Health Organization and caused widespread panic and anxiety among the public (World Health Organization, 2020a,b). However, there are potential opportunities in the crisis of the COVID-19 epidemic. The present study aimed to investigate the positive role of negative mood on creativity, revealing an opportunity in the crisis of the COVID-19 epidemic.

### The Negative Effect of the COVID-19 Epidemic

The outbreak of COVID-19 in 2020 has had a great impact on both society and individuals. According to Lazarus and Folkman’s (1984) theory of psychological stress, a crisis would induce a series of emotional, behavioral, and physiological stress responses in individuals. The widespread negative mood will inevitably have a severe impact on people’s mental health. In the face of the COVID-19 outbreak, the public would experience the fluctuation of emotional states and generally experienced negative moods such as anxiety, anger, helplessness, and panic (Wang J. et al., 2020). According to the word frequency of posts in Sina Weibo, a widely used Chinese social media site, a study revealed that negative moods (e.g., anxiety and anger) increased, happiness decreased, and sensitivity to social risks increased among the general public after the National Health Commission defined COVID-19 (see Zhu et al., 2020, for review).

### The Positive Effect of the COVID-19 Epidemic

#### Mood and Cognitive Creativity

In the current study, we attempted to further investigate the positive effect of negative mood on two areas of creativity: cognitive creativity and emotional creativity.

The negative mood we measured is a mood state that built up gradually in uncertain situations (during the COVID-19 outbreak), which is easy to control, less intense, and more persistent (Beedie et al., 2005). Cognitive creativity, also known as creative ideas or thinking, can generate creative ideas of alternative solutions to a problem (Guilford, 1967). Creative ideation (CI) refers to the process of generating original ideas in response to open-ended problems (Fink and Benedek, 2014). It is a universal component of creativity in that creativity at all levels involves ideation (Runco et al., 2001). Benedek et al. (2012) also suggested that the generation of new ideas is the core of every conception of creativity.

Numerous empirical studies have provided evidence for a relationship between mood and creative ideation. However, there is little consensus about what types of mood are the most conducive to the process of creative ideation (De Dreu et al., 2008; Davis, 2009). To clarify the mood–idea generation relationship, it is necessary to clarify the types of mood. First, according to valence, moods could be divided into positive mood and negative mood. A large number of studies generally believed that positive mood could promote creative ideation, while negative mood may hinder creative ideation (Hirt et al., 2008). However, some studies found that negative mood could also promote creative ideation, which might be due to the arousal of mood (Russ and Kaugars, 2001; De Dreu et al., 2011). Therefore, we could divide mood into low arousal moods (e.g., sad, depressed, relaxed, serene) and high arousal moods (e.g., angry, fearful, happy, and elated) according to their different arousal levels (De Dreu et al., 2008). Some researchers believed that high arousal mood could promote creative ideation, but it has nothing to do with the emotion valence (Tsai et al., 2013). However, this does not explain why patients with depression have long-term sadness and depression and have better performance in creativity (Jamison et al., 1980). Furthermore, Higgins (1997) put forward regulatory focus theory, that mood state could be regulated by the promotion motivational system and prevention motivational system. The promotion focus refers to entailing motivation to achieve one’s goal, and the prevention focus refers to entailing motivation to attain security (e.g., shelter from harm). Some researchers believed that the preventive-focused moods (e.g., fear, anxiety, etc.) could promote creative performance (Friedman and Förster, 2001; De Dreu et al., 2008). In addition, situations also affected the relationship between mood and creative ideation. Feist (1999) believed that, in uncertain situations or threats, individuals’ problems may cause negative moods, such as anxiety and tension. Meanwhile, this kind of negative mood in uncertain situations is likely to be a source of creativity (Runco, 1999). Similarly, many studies have shown that negative mood experiences could promote creative performance during the idea generation (Baas et al., 2008; Damian and Simonton, 2015). Other researchers believed that the negative mood would improve the originality and flexibility of creative ideation (Rahimi and Sabahi, 2019). It is possible that mild negative mood can enhance creativity by generating powerful thinking and strengthening perseverance (Baas et al., 2008; Lee et al., 2017). Based on the available evidence, we supposed that the negative mood during uncertain and risk situations may promote creative ideation.

#### Mood and Emotional Creativity

Emotional creativity is an ability to produce a new and appropriate emotional response and is an extension of cognitive creativity in the field of emotion (Averill, 1999). It has four evaluation factors: preparedness, novelty, effectiveness, and authenticity. Preparedness means that individuals, attached to emotions, are willing to think actively, understand, and explore emotions and are sensitive to other people’s emotions. Novelty means that the individual’s emotional responses are novel compared with their previous emotional reactions or typical emotions in society. Effectiveness means that the individual’s emotional responses play an essential role in solving the emotional problems, and, in the long run, it is beneficial to the individual and beneficial to the group and society. Authenticity refers to the individual’s emotional responses reflected in a certain way.

In particular, the influence of negative mood on emotional creativity can be explicated from two aspects: (1) the relationship between emotional creativity and cognitive creativity and (2) the effectiveness of emotional creativity. First, emotional creativity is an extension of cognitive creativity in the field of emotion (Averill, 1991). Secondly, the effectiveness is one of the criteria to evaluate emotional creativity (Averill, 1999), which refers to an emotional response that has potential value and benefits to a person or a group. Meanwhile, negative mood could prompt an individual to have a fast and adapted reaction (Fredrickson and Cohn, 2008). Therefore, we inferred that negative mood could promote emotional creativity by enhancing the effectiveness of emotional expression.

### Self-Focused Attention

Self-focused attention is the attention to self-related information (Ingram, 1990). It is the introspection of one’s thoughts and feelings. In our daily life, moderate self-focused attention may help individuals monitor and regulate their thoughts and behaviors, which helps improve negative mood and eliminate their influence on cognitive activities. However, a high degree of self-focused attention tends to interrupt the processing of environmental information and external tasks and affect individuals’ effective problem-solving process. Scheier and Carver’s (1977) “valence theory” suggested that self-focused attention increased emotional valence and enhanced emotional experience. However, other researchers found the opposite result and proposed “self-regulation theory,” showing that self-focused attention would reduce emotional feelings (McFarland et al., 2007).

These studies above showed that in some cases, self-focused attention led to negative mood outcomes, while in other cases, it had positive mood outcomes. Such a difference of self-focused attention led Trapnell and Campbell (1999) to propose two classifications of chronic attention to self: rumination and reflection. According to their classifications, rumination was mainly about one’s focus on the causes and consequences of difficulties rather than alleviating the stress of the problems. Reflection was characterized by an open exploration of negative mood, a feeling that one’s mood was clear and controllable, and a willingness to consider strategies to alleviate unpleasant moods. Baer (2007) believed that rumination was a frequent kind of thinking. Individuals would constantly ponder over personal problems and shortcomings and looked back on the wrong things that had happened and possible reasons. On the contrary, reflection can help individuals consciously think about ways to engage in problem-solving.

The relationship between rumination and reflection can be explained by the Analytical Rumination Hypothesis (ARH; Bartoskova et al., 2018), which suggests that negative mood promoted a two-stage meditation process. The first stage is to analyze the cause of the problem (causal analysis). The second stage is to analyze the solution (problem-solving analysis). The basic idea is that, in the scientific community, as a common solution, one must first understand a complex problem in order to solve it (Andrews and Thomson, 2009; Barbic et al., 2014). In Takano and Tanno’s (2009) study, they also found that rumination could predict reflection. Based on these findings, we speculated that rumination and reflection also have a relationship of one-causing-the-other.

Self-focused attention is inseparable from mood and linked to creativity through depression. Persistent negative mood or loss of interest was the character of depression as mood or affective disorders (Zhang et al., 2019). Our investigation on the relationships among depression, self-focused attention, and creativity would help us explain the relationships among negative mood, rumination, reflection, and creativity. Focusing on self and personal feelings can be an important part of creative activities, especially for writers and poets. Interestingly, in a study by Jamison et al. (1980), people with bipolar disorder reported that their depression state led to a heightened sensitivity to their mood, which in turn had a positive effect on their creativity. Kaufmann (2015) and Forgeard et al. (2020) have also found that negative mood was beneficial when identifying problems and evaluating ideas during creative thinking (for example, to measure the quality of one’s ideas). Supported by such evidence, other scholars believed that the stimulative effect of depression state in the creative process was due to the increased introspection, which led to high sensitivity to internal content (Richards, 1981). Richards (1981) explained that this form of introspection contributed to the content of the creative work that later emerged as a product of the creative process during functional improvement. Verhaeghen (2005, 2014) found that reflection played a critical role in the relationship between depressive symptoms and the creative performance. Depression and rumination are closely related. Forgeard et al. (2020) found that rumination can explain the relationship between depressive symptoms and the generation of more valuable ideas. This may be because the fact that the rumination made people set a high and strict standard for themselves, and people may use these same standards to produce thoughts.

Self-focused attention may also affect emotional creativity. When Trnka et al. (2019) studied the emotional creativity of the elderly, they found that the participants, who showed less focus on their emotional responses and searching fewer reasons for their feelings, also had lower scores in the preparedness component of the Emotional Creativity Inventory. Participants had higher scores in the preparedness component of the Emotional Creativity Inventory when they were more focused on their emotional responses and experiences compared with their less-focused counterparts. Meanwhile, in the process of self-focus, monitoring self-mood was the key to emotional intelligence (Mayer et al., 2001), which helped to improve negative mood (McFarland et al., 2007) and eliminate its negative influence on cognitive activities (Dunn and Schweitzer, 2005). Since previous studies have shown that adaptive emotion regulation was one of the manifestations of emotional creativity, we inferred that self-focused attention was associated with emotional creativity.

Though it has been observed that the two self-focused attention variables (i.e., rumination, reflection) were strongly associated with negative mood and creativity, there is no study available on the relationships among the two self-focused attention variables (i.e., rumination, reflection), cognitive creativity, and emotional creativity. Perhaps, self-focused attention here may provide a bridge between negative mood and creativity.

### Cognitive Creativity and Emotional Creativity

As mentioned above, previous studies have suggested that there might be a potential connection between cognitive creativity and emotional creativity. However there is an essential difference between them (Fuchs et al., 2007; Ivcevic et al., 2007; Furnham, 2016). First, cognitive creativity and emotional creativity have different meanings. Cognitive creativity is a crucial cognitive function that enables individuals to solve problems by generating novel and useful products or ideas (Ogawa et al., 2018). Emotional creativity is a dispositional trait that resulted from experiencing a complicated emotional life (Averill, 2009). Second, there are different evaluation criteria for cognitive creativity and emotional creativity. Cognitive creativity has two criteria: originality and usefulness (Runco and Garrett, 2012). Emotional creativity is evaluated by four criteria: preparedness, novelty, effectiveness, and authenticity (Averill, 1999). In Martsksvishvili (2017) study, they found that emotional creativity was not associated with the originality and appropriateness scores of the Alternative Uses Tasks (AUT; Guilford, 1950), which is an experimental paradigm commonly used to measure divergent thinking, when they examined the psychometric properties of the Georgian version of Emotional Creativity Inventory (G-ECI; Averill, 1999). In addition, emotional creativity did not play a significant role in the creative expression of divergent thinking (Zenasni and Lubart, 2008). Therefore, it has been inferred that emotional creativity was related to creative activities only when emotions are related to creative activities (Ivcevic et al., 2007). However, in general, emotional creativity is not related to creative achievements and creative thinking (Martsksvishvili, 2017). Because of these differences between cognitive creativity and emotional creativity, we inferred that they might have different relationships with the psychological impact of the COVID-19 outbreak.

### The Present Study

As discussed in the previous sections, the relationships among negative mood, self-focused attention, and creativity remain unclear; the difference between cognitive creativity and emotional creativity in the face of the psychological impact of the COVID-19 epidemic is still not clear. Based on the literature of the relationships among negative mood, rumination, reflection, creative ideation, and emotional creativity, we hypothesized that (H1) the negative mood during COVID-19 epidemic may influence creative ideation through the serial mediation of rumination and reflection; (H2) the negative mood during the COVID-19 epidemic may influence emotional creativity through the serial mediation of rumination and reflection; and (H3) cognitive creativity and emotional creativity have different relationships with the psychological impact of COVID-19 outbreak.

To explore these relationships, we collected and analyzed the data of 351 college students during the COVID-19 epidemic period. Through the design of the present study, we investigated whether negative mood during the epidemic could have a serious impact on individual mental health and play a positive role of negative mood on creativity under the serial mediation of rumination and reflection.

## Materials and Methods

### Participants

Three hundred and fifty-one college students at Shaanxi Normal University (57 males, 294 females, mean age: 19.30 ± 0.63 years, range: 18–21 years) were recruited for the present study. A convenient sampling technique was followed. All participants had normal or corrected to normal vision and had no history of neurological or psychiatric illness and substance abuse. None of the subjects had been infected with COVID-19. The study conformed to the principles of the Declaration of Helsinki (World Medical Association [WMA], 2013) and was approved by the Academic Committee of the MOE Key Laboratory of Modern Teaching Technology, Shaanxi Normal University in China. All participants signed a written informed consent form after the procedures were thoroughly explained, and they were paid for their participation in the current study.

### Measures

Participants finished a demographic survey, the Runco Ideational Behavior Scale (RIBS), the Emotional Creativity Inventory, the state-trait Anxiety Inventory (STAT), the Positive and Negative Affect Schedule (PANAS), the Impact of Event Scale-Revised (IES-R), and the Depression Subscale of Depress Anxiety Stress Scale 21 (DASS-21) through a widely used online survey platform^1^ on their cell phones. In this study, the tools we used to measure negative mood were the PANAS (Watson et al., 1988), DASS-21 (Lovibond and Lovibond, 1995), STAI (Spielberger, 1983), and IES-R (Christianson and Marren, 2012). However, we used the data of PANAS-neg to analyze the serial mediation effect. DASS-21, STAI, and IES-R were used to analyze the correlation and build the structural equation modeling for detecting the relationship between negative moods and creativity. Their corroborating with each other suggested that the data we collected about negative moods are valid, and the results of our study are stable and reliable.

Meanwhile, we also did some work to reduce bias during data collection. First, all investigators were trained for questionnaire investigation and data collection to reduce bias during data collection. Second, we emphasized in the instruction of the questionnaire survey: “You should answer the questions carefully and report your real thoughts. There are no right or wrong options.” Third, we set some questions in the questionnaire to check whether the subjects answered the questions carefully, such as forced selection (“please select a certain item”), repeat selection (“in accordance with a previous question”), and reverse scoring (some items were reversely coded and the total scores were used in analysis). Finally, we screened the collected data to eliminate the results of failed lie detection and abnormal answers (answers with regular patterns).

### The Runco Ideational Behavior Scale

The RIBS is used to assess the participants’ creative ideation. Creative ideation (CI) referred to the process of generating original ideas in response to given open-ended problems (Fink and Benedek, 2014). The RIBS (Runco et al., 2001) consists of 23 items that are measured on a 5-point Likert scale (1 = never and 5 = very often). This one-dimensional scale is based on the theory that certain ideas may be products of not only original or divergent thinking but creative thinking as a whole (Guilford, 1967). The internal consistency reliability of RIBS was satisfactory in the present study (Cronbach’s α = 0.86).

### The Emotional Creativity Inventory

The Emotional Creativity Inventory (ECI) is used to assess the participants’ emotional creativity. The ECI (Averill, 1999) consists of three factors: readiness (the ability to understand and learn the emotions of one and others), innovation (the ability to experience unusual emotions), efficiency, and honesty (the ability to express emotions skillfully and honestly), with a total of 30 items. At present, the ECI is widely used and has cross-cultural adaptability. The internal consistency reliability of ECI was satisfactory in the present study (Cronbach’s α = 0.80).

### The Rumination–Reflection Questionnaire

The Rumination–Reflection Questionnaire (RRQ) is used to measure the two factors of self-focused attention (i.e., rumination and reflection). The RRQ (Trapnell and Campbell, 1999) has two subscales and consists of 24 items. Each subscale consists of 12 items that are measured on a 5-point Likert scale (1 = totally disagree, 2 = slightly disagree, 3 = neutral, 4 = somewhat agree, 5 = completely agree). The higher the score, the greater the tendency of the subject in this dimension (Yuan et al., 2010).

The internal consistency reliability of RRQ was satisfactory in the present study (Cronbach’s α = 0.87; for rumination subscale, Cronbach’s α = 0.84; for reflection subscale, Cronbach’s α = 0.87).

### The State-Trait Anxiety Inventory

The STAI is used to assess the participants’ state anxiety. The 40-item State-Trait Anxiety Scale (Spielberger, 1983) consists of two subscales: state anxiety (STAI-Formy-1 for the first 20 items) and trait anxiety (STAI-Formy-2 for the last 20 items), using a four-point scale. The state anxiety subscale used in this study is generally used to assess current or recent experiences and feelings of panic, anxiety, or nervousness about a situation at a particular time. Half of the items described positive mood (reverse scoring), and half described negative mood (positive scoring), with higher scores indicating higher levels of state anxiety. The internal consistency reliability of STAI-Formy-2 was satisfactory in the present study (Cronbach’s α = 0.90).

### The Positive and Negative Affect Schedule

The PANAS is used to assess the participants’ positive and negative affect. The 20-item PANAS (Watson et al., 1988) consists of two subscales: positive affect (PANAS-pos) and negative affect (PANAS-neg). The current study only used the PANAS-neg component to assess negative affect. Higher scores on PANAS-neg indicated higher negative disposition or affect. The internal consistency reliability of PANAS was satisfactory in the present study (Cronbach’s α = 0.84; for PANAS-neg, Cronbach’s α = 0.89).

### The Impact of Event Scale-Revised

The impact of event scale (IES-R) is used to measure and assess the participants’ catastrophic experience of a particular life event, with the addition of a hyper-alert component (reviewed by Christianson and Marren, 2012). The 22-item IES-R (developed by Horowitz et al., 1979; revised by Weiss and Marmar, 1997) consists of three subscales: invasive symptoms, avoidance symptoms, and high arousal symptoms. In our study, we used it to measure the psychological impact of the COVID-19 epidemic.

The IES-R has been well-validated in the Chinese population for determining the extent of psychological impact after exposure to a public health crisis within 1 week of exposure (Zhang, 2014; Wang C. et al., 2020). The specific life event in this study was the COVID-19 epidemic. The total IES-R score is divided into 0–23 (normal), 24–32 (mild psychological impact), 33–36 (moderate psychological impact), and > 37 (severe psychological impact) (Creamer et al., 2003). The internal consistency reliability of IES-R was satisfactory in the present study (Cronbach’s α = 0.94).

### The Depression Subscale of Depress Anxiety Stress Scale 21

The complete version of the depress anxiety stress scale (DASS) is used to assess the participants’ depression, anxiety, and stress. The DASS (compiled by Lovibond and Lovibond, 1995) consists of three subscales: depression, anxiety, and stress, with 42 items in total. In this study, the simplified version of DASS (DASS-21) revised by Antony et al. (1998) and Crawford and Henry (2003) were adopted. The guidance emphasized, “Please follow your situation in the last week.” Studies showed that DASS-21 had the same factor structure and the same reliability and validity as the full version. The internal consistency reliability of DASS-21 was satisfactory in the present study (Cronbach’s α = 0.94; for stress subscale, Cronbach’s α = 0.88; for anxiety subscale, Cronbach’s α = 0.83; for depression subscale, Cronbach’s α = 0.85).

### Statistical Procedures

The descriptive statistics for variables were computed. Then, bivariate correlations were calculated to test the relationships among the variables included in the serial mediation analyses.

All data used in this study were calculated using IBM Statistical Package for Social Sciences (SPSS) version 23.0 (SPSS Inc., Chicago, United States) and PROCESS version 3.5 macro for SPSS were used to analyze the data (Preacher and Hayes, 2004; Hayes, 2013). The serial mediation effect analysis was tested using the Model 6 and bootstrap method by running the PROCESS plugin in the SPSS software. The SEM was implemented in *AMOS* 23.0 (SPSS Inc., Chicago, United States).

## Results

As our survey was self-reported, common method bias was tested. First, we checked the values of correlation coefficients among contracts in Tables 1, 2 to see whether they are too high (i.e., *r* > 0.90). It was found that all the values were not beyond the threshold (Pavlou and Sawy, 2006). Second, we conducted a Harman single-factor test using the principal component analysis. The results showed that one factor explained 16.056% of the variance, an index of less than 50% (Podsakoff and Organ, 2016). Our results also confirmed that no single factor accounted for the majority of covariance. Therefore, non-response bias and common method bias did not affect the results and the findings in the current study.

**TABLE 1:** The correlations between negative mood, state anxiety, depression, rumination, reflection, and the IES-R scores.

	**Negative mood**	**State anxiety**	**Depression**	**Stress**	**Anxiety**
The IES-R scores	0.436***	0.381***	0.482***	0.619***	0.540***
Rumination	0.303***	0.239***	0.248***	0.290***	0.245***
Reflection	0.168**	−0.028	0.014	0.064	0.063

***p < 0.001, **p < 0.01.

**TABLE 2:** The correlations between negative mood, self-focused attention (i.e., rumination, reflection), cognitive creativity, and emotional creativity.

	**1**	**2**	**3**	**4**	**5**
1 Negative mood	1				
2 Rumination	0.303***	1			
3 Reflection	0.168**	0.298***	1		
4 Creative ideation	0.158**	0.182***	0.419***	1	
5 Emotional creativity	0.217***	0.251***	0.456***	0.584***	1

***p < 0.001, **p < 0.01.

The psychological impact of the COVID-19 epidemic, measured using the IES-R scale, was averaged 21.40 (*SD* = 14.29). Among all participants, 199 (56.7%) reported minimal psychological impact (score < 23); 78 (22.2%) rated mild psychological impact (scores 24–32); and 74 (21.1%) reported a moderate or severe psychological impact (score > 33). The IES-R scores were positively correlated with negative mood (*r* = 0.436, *p* < 0.001), state anxiety (*r* = 0.381, *p* < 0.001), stress (*r* = 0.619, *p* < 0.001), anxiety (*r* = 0.540, *p* < 0.001), and depression (*r* = 0.482, *p* < 0.001) during the COVID-19 epidemic. These results indicated that the data we measured on negative mood were associated with the COVID-19 epidemic and had general applicability. These results had good ecological validity. At the same time, we can also see that rumination was positively correlated with negative mood (*r* = 0.303, *p* < 0.001), state anxiety (*r* = 0.245, *p* < 0.001), stress (*r* = 0.290, *p* < 0.001), anxiety (*r* = 0.248, *p* < 0.001), and depression (*r* = 0.248, *p* < 0.001), while reflection was positively correlated with negative mood (*r* = 0.168, *p* = 0.002) (Table 1).

### The Positive Effects of Negative Mood on Creativity

#### Correlation Analysis

Table 2 represents the correlations among the variables during the COVID-19 epidemic. The results showed that negative mood was correlated with rumination (*r* = 0.303, *p* < 0.001), reflection (*r* = 0.168, *p* = 0.002), creative ideation (*r* = 0.158, *p* = 0.003), and emotional creativity (*r* = 0.217, *p* < 0.001). Rumination was associated with reflection (*r* = 0.298, *p* < 0.001), creative ideation (*r* = 0.182, *p* = 0.001), and emotional creativity (*r* = 0.251, *p* < 0.001). Reflection was associated with creative ideation (*r* = 0.419, *p* < 0.001) and emotional creativity (*r* = 0.456, *p* < 0.001). Creative ideation was correlated with emotional creativity (*r* = 0.584, *p* < 0.001). In the serial mediation model of the present study, we predicted that negative mood was the independent variable; rumination and reflection were potential mediators. Creative ideation and emotional creativity were dependent variables. The correlations found among those variables would be the basis of following serial mediation analysis.

### Serial Mediation Analysis

#### Cognitive Creativity

The serial mediation analysis was carried out based on H1: the negative mood during COVID-19 epidemic may influence creative ideation through the serial mediation of rumination and reflection.

**FIGURE 1:**
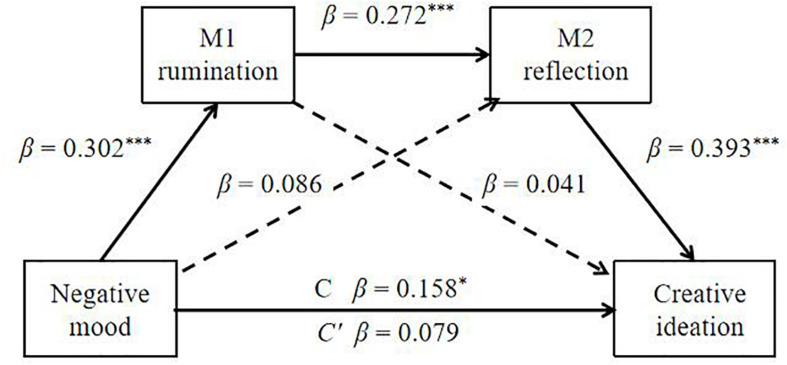
Serial mediation model (Model 1) of rumination and reflection on the relationship between negative mood and creative ideation. The statistical result is satisfied for a mediation effect: negative mood (X) influences creative ideation (Y) through rumination (M1) and reflection (M2). The direct path from negative mood to reflection and the direct path from rumination to creative ideation were not significant. β: standardized coefficients. ***p < 0.001, *p < 0.05.

The serial mediation model was computed with two mediators (Model 1; M1: rumination and M2: reflection; Figure 1). The total effect (β = 0.158, *p* = 0.031) from negative mood to creative ideation was at a significant level. Moreover, the direct path from negative mood to rumination (M1) (β = 0.302, *p* < 0.001) was significant. Meanwhile, the path from the first mediator (M1: rumination) to the second mediator (M2: reflection) was also significant (β = 0.272, *p* < 0.001). The path from the mediator, namely, reflection (β = 0.393, *p* < 0.001), to creative ideation was significant. However, the path from another mediator, rumination (β = 0.041, *p* = 0.433), to creative ideation was not significant. Meanwhile, the direct paths from negative mood to reflection (β = 0.086, *p* = 0.108) and creative ideation (β = 0.079, *p* = 0.123) were not significant. Moreover, the mediators (rumination and reflection) were observed to exert a serially mediated effect on the relationship between negative mood and creative ideation.

Table 3 shows the indirect effects and their associated 95% CIs. As shown in the table, the analysis of the indirect mediation by bootstrapping found that the resulting data supported the significance of Path 3 [Negative mood → Rumination → Reflection → Creative ideation, β = 0.032, *SE* = 0.012, 95% *CI* (0.012, 0.060)]. However the Path 1 [Negative mood → Rumination → Creative ideation; β = 0.013, *SE* = 0.016, 95% *CI* (−0.017, 0.046)] and Path 2 [Negative mood → Reflection → Creative ideation; β = 0.034, *SE* = 0.022, 95% *CI* (−0.008, 0.079)] were not significant. Therefore, the effect of negative mood on creative ideation was realized through serial mediation of rumination and reflection.

**TABLE 3:** Bootstrapping indirect effects and 95% confidence intervals (CI) for the final mediational model.

	**Model pathways**	**β**	**95%*CI***	
			**Lower**	**Upper**
	Total indirect effect	0.078	0.029	0.131
Path1	Negative mood → Rumination → Creative ideation	0.013	−0.017	0.046
Path2	Negative mood → Reflection → Creative ideation	0.033	−0.008	0.079
Path3	Negative mood → Rumination → Reflection → Creative ideation	0.032	0.012	0.059

*β = standardized coefficients.*

**FIGURE 2:**
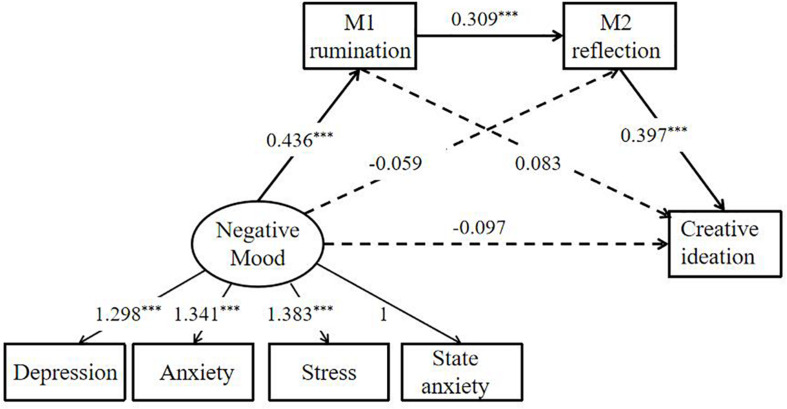
Structural equation model (Model 2) depicts regression paths in this serial mediation model. Large circles represent latent variables; rectangles represent single-item indicators; and single-headed arrows represent regression coefficients. Regression coefficients are standardized, ***p < 0.001.

Furthermore, to further examine whether our findings were stable and reliable, we also used the data from DASS-21 and STAI-Formy-2 to build a structural equation modeling (SEM) to test H1. We created the SEM with the “Negative Mood” as the latent variable and the depression, anxiety, stress, and state anxiety as the manifest variables. Following our H1, we presumed that rumination and reflection are the mediators in the association between negative moods (depression, anxiety, stress, and state anxiety) and creative ideation. In other words, the negative mood during COVID-19 epidemic may influence creative ideation through the serial mediation of rumination and reflection. The model fit the data quite well [CMIN/DF = 1.858 (*p* = 0.040), GFI = 0.984, IFI = 0.992, TLI = 0.985, RMSEA = 0.050]. As shown in Figure 2 (Model 2), the analysis of the indirect mediation by 5,000 bootstrapping supported the significance of Path 3 [Negative Mood → Rumination → Reflection → Creative ideation; β = 0.054, *SE* = 0.020, 95% *CI* (0.026, 0.092)]. However, the Path 1 [Negative Mood → Rumination → Creative ideation; β = 0.036, *SE* = 0.028, 95% *CI* (−0.002, 0.087)] and Path 2 [Negative Mood → Reflection → Creative ideation; β = −0.023, *SE* = 0.036, 95% *CI* (−0.080, 0.040)] were not significant.

The two serial mediation models (Model 1 and Model 2) were consistent with the finding that negative mood during the COVID-19 epidemic could promote an individual’s creative ideation through serial mediation of rumination and reflection. These results implied that the DASS-21, the State anxiety, and the PANAS-neg were corroborated with each other, and the results of our study were stable and reliable.

#### Emotional Creativity

**FIGURE 3:**
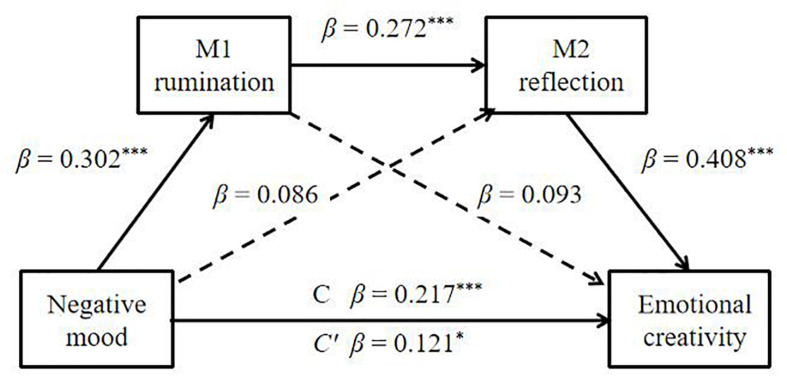
Serial mediation model (Model 3) of the rumination and reflection on the relationship between negative mood and emotional creativity. The statistical result is satisfied for a mediation effect: negative mood (X) influences emotional creativity (Y) through rumination (M1) and reflection (M2). The direct path from negative mood to reflection and the direct path from rumination to emotional creativity were significant. β: standardized coefficients, ***p < 0.001, *p < 0.05.

The serial mediation analysis was carried out based on H2: the negative mood during COVID-19 epidemic may influence emotional creativity through the serial mediation of rumination and reflection. The serial mediation model was computed with two mediators (Model 3; M1: rumination, M2: reflection; Figure 3). The total effect (β = 0.217, *p* < 0.001) from negative mood to emotional creativity was significant. The direct path from negative mood to emotional creativity (β = 0.121, *p* = 0.015) was significant. Moreover, the direct path from negative mood to rumination (M1) (β = 0.302, *p* < 0.001) was significant. Meanwhile, the path from the first mediator (M1: rumination) to the second mediator (M2: reflection) was also significant (β = 0.272, *p* < 0.001). The path from the mediator, namely, reflection (β = 0.408, *p* < 0.001), to emotional creativity was significant. However, the path from another mediator, rumination (β = 0.093, *p* = 0.068), to emotional creativity was not significant. Meanwhile, the direct path from negative mood to reflection (β = 0.086, *p* = 0.108) was not significant. Moreover, the mediators (rumination and reflection) were observed to exert a serial mediation effect on the relationship between negative mood and emotional creativity.

Table 4 shows the indirect effects and their associated 95% *CI*s. As shown in the table, the analysis of the indirect mediation by bootstrapping found that the resulting data supported the significance of Path 3 [Negative mood → Rumination → Reflection → Emotional creativity; β = 0.0335, *SE* = 0.012, 95% *CI* (0.013, 0.060)]. However, Path 1 [Negative mood → Rumination → Emotional creativity; β = 0.028, *SE* = 0.018, 95% *CI* (−0.002, 0.069)] and Path 2 [Negative mood → Reflection → Emotional creativity; β = 0.035, *SE* = 0.023, 95% *CI* (−0.008, 0.081)] were not significant. Thus, the effect of negative mood on emotional creativity was realized through serial mediation of rumination and reflection.

**TABLE 4:** Bootstrapping indirect effects and 95% confidence intervals (CI) for the final mediational model.

	**Model pathways**	**β**	**95%*CI***	
			**Lower**	**Upper**
	Total indirect effect	0.096	0.046	0.154
Path1	Negative mood → Rumination → Emotional creativity	0.028	−0.002	0.069
Path2	Negative mood → Reflection → Emotional creativity	0.035	−0.008	0.080
Path3	Negative mood → Rumination → Reflection → Emotional creativity	0.034	0.013	0.060

*β = standardized coefficients.*

**FIGURE 4:**
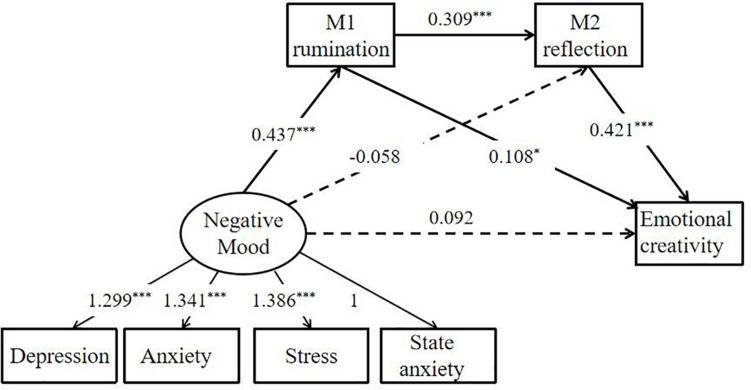
Structural equation model (Model 4) depicts regression paths in this serial mediation model. Large circles represent latent variables; rectangles represent single-item indicators; single-headed arrows represent regression coefficients. Regression coefficients are standardized, ***p < 0.001, *p < 0.05.

We also created the SEM with the “Negative Mood” as the latent variable and the data from DASS-21 and STAI-Formy-2 as the manifest variables to test the proposed relationship between negative mood and emotional creativity. Following our H2, we presumed that rumination and reflection are two mediators of the association between the negative moods (depression, anxiety, stress, and state anxiety) and emotional creativity. In other words, the negative mood during COVID-19 epidemic may influence emotional creativity through the serial mediation of rumination and reflection. The model fit the data quite well [CMIN/DF = 1.576 (*p* = 0.098), GFI = 0.986, IFI = 0.995, TLI = 0.990, RMSEA = 0.041]. As shown in Figure 4 (Model 4). The analysis of the indirect mediation by 5,000 bootstrapping supported the significance of Path 3 [Negative Mood → Rumination → Reflection → Emotional creativity, β = 0.057, *SE* = 0.021, 95% *CI* (0.029, 0.099)]. Path 1 [Negative Mood → Rumination → Emotional creativity; β = 0.047, *SE* = 0.030, 95% *CI* (0.006, 0.104)] was marginally significant. However Path 2 [Negative Mood → Reflection → Emotional creativity; β = −0.025, *SE* = 0.038, 95% *CI* (−0.086, 0.040)] was not significant.

The two serial mediation models (Model 3 and Model 4) were consistent with the finding that negative mood during the COVID-19 epidemic could promote an individual’s emotional creativity through serial mediation of rumination and reflection. These findings implied that the DASS-21, the State anxiety, and the PANAS-neg were corroborated with each other and the results of our study were stable and reliable.

### The Different Impact on Cognitive Creativity and Emotional Creativity

Table 5 shows that the IES-R scores were positively correlated with emotional creativity (*r* = 0.118, *p* = 0.027) and were not related to cognitive creativity (*r* = 0.082, *p* = 0.127).

**TABLE 5:** The correlations between cognitive creativity, emotional creativity, and the IES-R scores.

	**1**	**2**	**3**
1 The IES-R scores	–	0.082	0.118*
2 Cognitive creativity		–	0.584***
3 Emotional creativity			–

***p < 0.001, *p < 0.05.

**FIGURE 5:**
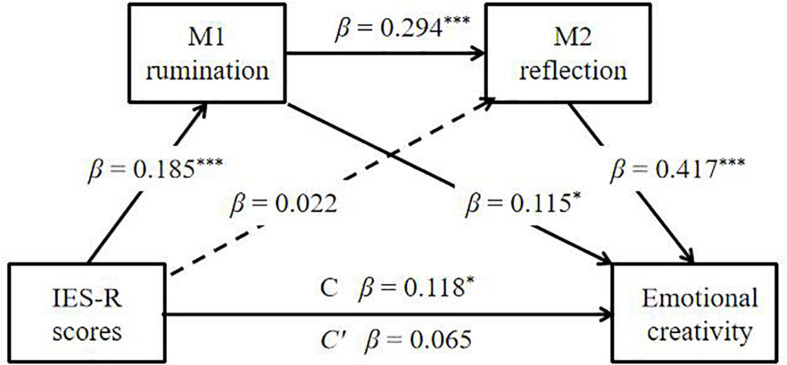
Serial mediation model (Model 5) of the rumination and reflection on the relationship between IES-R scores and emotional creativity. The statistical result is satisfied for a mediation effect: IES-R scores (X) influence emotional creativity (Y) through rumination (M1) and reflection (M2). β: standardized coefficients. ***p < 0.001, *p < 0.05.

Meanwhile, Figure 5 shows that the mediators (i.e., rumination and reflection) were observed to exert a serial mediation effect on the relationship between the psychological impact of the COVID-19 epidemic (IES-R scores) and emotional creativity. The analysis of the indirect mediation by 5,000 bootstrapping supported the significance of Path 3 [IES-R scores → Rumination → Reflection → Emotional creativity; β = 0.023, *SE* = 0.010, 95% CI (0.006, 0.045)]. Path 1 [IES-R scores → Rumination → Emotional creativity; β = 0.021, *SE* = 0.013, 95% *CI* (0.001, 0.054)] was marginally significant. However, Path 2 [IES-R scores → Reflection → Emotional creativity; β = 0.091, *SE* = 0.025, 95% *CI* (−0.039, 0.058)] was not significant. Thus, the effect of the psychological impact of the COVID-19 epidemic (IES-R scores) on emotional creativity was realized through serial mediation of rumination and reflection. However, the psychological impact of the COVID-19 epidemic was not related to cognitive creativity. These results implied that the psychological impact of the COVID-19 epidemic and the PANAS-neg were corroborated with each other. Meanwhile, the COVID-19 epidemic had a different impact on cognitive creativity and emotional creativity.

## Discussion

In this study, we investigated the relationships among negative mood, rumination, reflection, cognitive creativity, and emotional creativity. We further demonstrated how rumination and reflection explained relationships between negative mood and creativity. Our analyses showed that (1) negative mood during the COVID-19 epidemic could promote the individual’s creative ideation and emotional creativity through serial mediation of rumination and reflection. (2) At the same time, the psychological impact after exposure to the COVID-19 epidemic was positively correlated with emotional creativity but not with creative ideation. We also illustrated the difference between cognitive creativity and emotional creativity. These results showed that the COVID-19 epidemic is a serious challenge and an opportunity for enhancing creativity in individuals.

### The Two-Sided Effect of the COVID-19 Epidemic

The data showed that negative mood during the epidemic had a negative impact on individuals. This result was consistent with the findings of the previous studies on the public psychological states during the COVID-19 epidemic (Wang J. et al., 2020), and recent articles urging people influenced by the epidemic to receive mental health care (Mei et al., 2011).

The collected data also showed there were different relationships among the mood variables (i.e., negative mood, state anxiety, stress, anxiety, and depression), rumination and reflection, respectively. Rumination had positive correlations with negative mood, state anxiety, stress, anxiety, and depression, while reflection was only associated with negative mood. The results were consistent with the previous studies. Takano and Tanno (2009) found that rumination was associated with a high level of depression, and reflection was associated with a low level of depression. Silvia et al. (2005) found that rumination was positively associated with negative mood; meanwhile, reflection may also not be entirely adaptive (Joormann et al., 2006). Miranda and Nolen-Hoeksema (2007) found that reflection prospectively predicted suicidal ideation. This relationship did not change after adjusting for depression severity and this may be related to the individual negative cognitive biases.

Moreover, the serial mediation analysis supported our two hypotheses. H1: the negative mood during the COVID-19 epidemic may influence creative ideation through the serial mediation of rumination and reflection. H2: the negative mood during COVID-19 epidemic may influence emotional creativity through the serial mediation of rumination and reflection. McFarland et al. (2007) found that negative mood could stimulate more self-related thoughts than neutral or positive mood and make individuals more likely to generate ruminative and reflective thinking. Kaufman and Baer (2002) also hold the same opinion in their study of depressed patients. Furthermore, we found that rumination and reflection were interrelated since they all belong to the two self-focused attention variables. This finding was in line with previous studies, which suggested that the reason for the interrelation between rumination and reflection might be due to their deeper connection to the behavioral inhibition system (Verhaeghen, 2014). Therefore, rumination can impair mood but does not affect creative thinking while reflection can enhance creative interests and creative originality (Verhaeghen, 2005).

Our study showed that creative ideation could be improved through serial mediation of rumination and reflection. One of the possible explanations may be that an individual in the rumination generated many thoughts. This would lead to two important results for creative ideation. The originality of ideas would appear when an individual has many thoughts (Albert, 1975; Simonton, 1994). Evidence also suggested that the speed of idea generation was proportional to the number of idea generation (Simonton, 1997). Such evidence implied that individuals with many thoughts may also have better performance in fluency. Our results suggested it is an opportunity to freely explore negative mood and the thoughts generated in the process of rumination while necessary strategies are also needed to control unpleasant moods. This was beneficial to promote cognitive creativity and emotional creativity.

Another possible explanation is that rumination could predict reflection and the two variables may be interdependent (Takano and Tanno, 2009). Self-focused attention would exacerbate the negative mood of individuals who were already in a state of dysphoric mood (Lyubomirsky and Nolen-Hoeksema, 1995). Negative mood would promote an analytical processing style (Forgas, 2013). This may be due to the fact that the individual would ruminate in negative mood while thinking about the cause of the problem (causal analysis) and then begin to analyze the problem (problem-solving analysis) in the reflective process triggered by rumination. Some scholars used this method to relieve depression (Hayes et al., 2005, 2007). To summarize, these scholars showed that the synergic effect of rumination and reflection could promote individual creative ideation.

### Cognitive Creativity and Emotional Creativity

We found that negative events (the COVID-19 epidemic) had a certain impact on the individual’s psychological states. The results of the correlations among cognitive creativity, emotional creativity, and the IES-R scores supported our third hypothesis: (H3) the IES-R scores positively correlated with emotional creativity but not with cognitive creativity. These results were consistent with Orkibi and Ram-Vlasov (2019), who suggested that there might be different mechanisms in emotional creativity and cognitive creativity.

In some cases, the best creative performance may require highly detail-oriented elaboration and analysis (Schwarz, 1989; Mackie and Worth, 1991). The products of cognitive creativity may originate from the dynamic interaction among the individual cognitive factors (information processing), dynamic (personality and motivation, etc.) and emotional factors (emotional states or traits), and the environment to stimulate or inhibit creativity potential (expression) (Barbot et al., 2011). Cognitive creativity needs more cognitive resources. However, the psychological impact of the COVID-19 epidemic inhibited individuals from extracting beneficial values from their thoughts, disrupted their use of information to control and regulated their thoughts and behaviors (Lee et al., 2017), and failed to form effective cognitive creative products. Therefore, the psychological impact of COVID-19 outbreak was not related to cognitive creativity.

The correlation between the psychological impact of the COVID-19 epidemic and emotional creativity suggested that the more the people were affected by negative events, the higher their scores of emotional creativity. This may be because the product of emotional creativity was an adaptive emotional response that points to self (Averill, 1991). Meanwhile, the adverse or traumatic experiences may generate tension and imbalance, which in turn may intrinsically motivate an individual to move toward creative adaptation through new cognitive and emotional reinterpretations of the experience (Runco, 1999). Other studies supported such a theory by showing that exposure to adverse experiences may enable individuals to generate unconventional emotional responses (Damian and Simonton, 2015; Damian, 2017). Averill (1999) also pointed out that previous traumatic experiences may lead one to think and be more innovative in emotional responses, which was conducive to the change of emotional suitability.

As discussed above, cognitive creativity is different from emotional creativity, and one of the differences between cognitive creativity and emotional creativity lies in the creative products of different categories of creativity. This is probably why the results showed that the psychological impact of the COVID-19 epidemic positively correlated with emotional creativity but not with cognitive creativity.

### Implication, Limitation, and Future Research

The results of the present study had relevant practical implications. First, as an unavoidable emotional experience in daily life, the positive roles of negative mood should be emphasized. For example, the current study suggested that we could achieve better creative performance self-focus in real life and work. Second, self-focused attention should be emphasized, especially the influence of reflection on cognitive creativity and emotional creativity. Such influence is important for negative mood to play a positive role in creativity. Therefore, in our daily life, education should pay more attention to reflection. As suggested by previous studies (Kross and Ayduk, 2008; Kross et al., 2011), it is also beneficial to include positive interventions (such as mindfulness) to improve self-focused attention in daily life, especially reflective thinking.

The study had some limitations as well as suggestions for future research. First, the cross-sectional design of the current study did not determine causality. In future studies, longitudinal and experimental methods can be used to analyze the causal relationships among negative mood, self-focused attention, and creativity. Second, most of our subjects were women. Future research should increase the sample size of male subjects for gender balance and gender effect analysis. Third, the sample in the current study was only Chinese college students. In further research, it is necessary to test the generalizability of our results across population samples. Forth, several studies suggested that mood defined in terms of mere valence could not explain the comprehensive relationship between creativity and negative mood (Baas et al., 2008; Davis, 2009; Rooij and Jones, 2013). In future research, we suggest further explore how different constituents of moods would influence creativity by inducing different mood states.

## Data Availability Statement

The raw data supporting the conclusions of this article will be made available by the authors, without undue reservation, to any qualified researcher.

## Ethics Statement

The studies involving human participants were reviewed and approved by the Academic Committee of the MOE Key Laboratory of Modern Teaching Technology, Shaanxi Normal University in China. The patients/participants provided their written informed consent to participate in this study. Written informed consent was obtained from the individual(s) for the publication of any potentially identifiable images or data included in this article.

## Author Contributions

YD designed the study, collected data, performed the data analysis, and drafted the manuscript. YY drafted, reviewed, and revised the manuscript. XW organized participants’ recruitment and analyzed data. CX and CL proofread the manuscript and validated the results. WH organized and funded the study. YL supervised the study and reviewed and proofread the manuscript. All authors contributed to the article and approved the submitted version.

## Conflict of Interest

The authors declare that the research was conducted in the absence of any commercial or financial relationships that could be construed as a potential conflict of interest.

## Footnotes

1 http://www.wjx.cn
